# Advances in Clinical Research on Traditional Chinese Medicine Treatment of Chronic Fatigue Syndrome

**DOI:** 10.1155/2020/4715679

**Published:** 2020-12-02

**Authors:** Xiaoyan Zhang, Miao Wang, Shigao Zhou

**Affiliations:** Longhua Hospital, Shanghai University of Traditional Chinese Medicine, Shanghai 200032, China

## Abstract

Chronic fatigue syndrome (CFS) is one of the most common chronic diseases in modern society and affects patients' quality of life to a certain extent. To date, the etiology and pathogenesis of CFS are still not completely clear. Various therapies have been developed, but there is still a lack of specific drugs or treatments. As a kind of adjuvant therapy, traditional Chinese medicine (TCM) has aroused widespread concern about the improvement of CFS. Although a large number of clinical randomized controlled trials have confirmed the therapeutic effect of TCM on CFS, the exact efficacy is still controversial. This article summarizes the clinical research methods and efficacy of TCM in the treatment of CFS over the past five years from the perspectives of syndrome differentiation, external treatment, and combination therapy.

## 1. Introduction

Since researchers led by the US Centers for Disease Control and Prevention coined the term Chronic fatigue syndrome (CFS), the history of the disease can be traced back to approximately 30 years ago [1]. In 2018, Nature announced the ten highest concerns in The Long-Term Special Report of Great Science, including Restarting the Study of CFS, whose influence cannot be underestimated [2]. CFS, or myositis encephalomyelitis (ME), is a multisystem chronic disease. It is a syndrome of debilitating diseases composed of fatigue, pain, cognitive impairment, and sleep disorders. To a certain extent, it can affect patients' quality of life [3, 4]. Some early studies showed that the proportion of the UK and American population suffering from CFS is between 0.2% and 2.6% [5, 6]. In 2019, Unger et al. [7] estimated that the prevalence rate in the Chinese CFS population was approximately 1%, which is basically consistent with the response data of epidemiological studies in Japan, Britain, and the United State.

## 2. Literature Collection and Collation

### 2.1. Literature Retrieval Strategy

The PubMed (2010～2020.3), Web of Science (2010～2020.3), JAMA Network (2015～2020.3), CNKI (2015～2020.3), SinoMed (2015～2020.3), and WanFang Data (2015～2020.3) electronic databases were searched. References were retrieved from the included documents. The Chinese search terms included *chronic fatigue syndrome*, *Chinese medicine treatment*, and *clinical trials*. The English search terms included *chronic fatigue syndrome, diagnosis,* and *treatment*. The combined searching of subject words and free words was used as the search strategy. There were no language restrictions for document retrieval.

### 2.2. Literature Inclusion and Exclusion Criteria

(1) Clinical research literature related to the treatment of chronic fatigue syndrome with traditional Chinese medicine, clear diagnostic criteria and evaluation criteria for efficacy, complete treatment processes, and affirmative efficacy was selected. Literature about the progress of traditional Chinese medicine in the treatment of chronic fatigue syndrome and literature related to the treatment of chronic fatigue syndrome was also reviewed. (2) Duplicates or literature abstracts, meeting minutes, guidelines, regulations, government reports, animal experiments, unpublished theses, and other document syndromes were removed from the documents retrieved from the above document source databases according to the prescribed search strategy. According to the title of the article and the title of the author, the reference documents retrieved were removed from duplicate documents.

### 2.3. Literature Search Results

In total, 331 articles were initially detected, and the two authors independently read the titles and abstracts, excluding 195 articles that did not meet the inclusion criteria. They further read the full text, screened the relevant literature of the original study as a randomized controlled trial, and finally included 77 articles, with 20 in English and 57 in Chinese.

## 3. Different Views between Modern Medicine and TCM on CFS

To date, the etiology and pathogenesis of CFS are still not completely clear. Modern medical considerations of the etiology and pathogenesis of CFS may be related to various factors, such as the immune and adrenal systems, genetic predisposition and certain personality traits, the neuroendocrine system, physical and emotional stress, biopsychosocial models, viral infections, childhood stress trauma, female gender, sleep quality, and nutrition [8–14]. There is also controversy in diagnosis. The Centers for Disease Control and Prevention (CDC) first proposed the diagnostic criteria for CFS in 1988 [15] and revised it in 1994, and the latter is used widely today. The definition of CFS depends on continuous or recurrent chronic fatigue lasting for more than 6 months. In addition, there must be 8 secondary standard symptoms [16]. The IOM published a research report in February 2015 suggesting that the disease should be renamed “systemic exertion intolerance disease” (SEID) and proposed a new diagnostic criterion indicating that patients must also have the following symptoms: (1) severe persistent (deep) fatigue that lasts for more than 6 months and cannot be relieved after rest (excluding other diseases or injuries); (2) discomfort that is significantly increased after exercise (postexertional malaise); and (3) unrefreshing sleep that cannot be eliminated after awakening. In addition to the above, at least one of the following two manifestations must also be present: (1) cognitive impairment or (2) orthostatic intolerance (meaning that the individual cannot stand for a long time). However, experts do not regard this as the best definition of CFS. It is generally considered that CFS can be treated by comprehensive methods such as medicine, nutrition, exercise, or psychology [17, 18]. Due to the lack of a specific therapy, many patients are still not able to recover and even worsen after taking various treatments, which wastes considerable time and money. It is difficult to find a therapy suitable for individuals.

TCM is a good choice as a therapy for CFS because of its long clinical history and universal efficacy. Although no exact records can be found in books, descriptions of fatigue and similar symptoms are equal to the records of “fatigue,” “emaciated”, “emptiness”, “slackness”, “exhaustion of limbs”, and “not lifting limbs” discussed by TCM [19]. Zhang [20] in the Han Dynasty first described chronic fatigue in *The Golden Chamber*, which is thought to be constituted as follows [21]: (1) insufficient endowment and weak constitution, (2) irregular work and rest, (3) disturbance by external factors or damage to the five internal organs, (4) infertility after illness, and (5) improper treatment and long-term strain. The pathogenesis is mainly the deficiency of the functions of the five internal organs, and the body loses Qi and blood. This description is accompanied by corresponding records of TCM syndrome differentiation and prescriptions. Some modern TCM scholars believe that the prevalence of CFS may be related to the physical constitution or emotional factors. Li et al. [22] analyzed the physical characteristics of CFS patients and found that Qi deficiency (32.4%), Qi depression (20.2%), Phlegm and Dampness Qualitative (17.6%) accounted for the top 3 in the entire patient population, and the characteristics of peace quality (0%) and special quality (0.5%) were very rare. Shen et al. [23] found that mental disorder is the causative factor of CFS and its accompanying symptoms. Corresponding TCM therapies are based on syndrome differentiation. In addition, TCM has a wealth of external therapies from which to choose. A large number of studies have shown that the combination of therapies of TCM can be more advantageous in the treatment of CFS and cause fewer adverse reactions. At present, the main clinical efficacy evaluation criteria of TCM effects on CFS include but are not limited to the fatigue scale-14 (FS-14), Fatigue Assessment Index (FAI), self-rating anxiety scale (SAS), self-rating depression scale (SDS), Pittsburgh Sleep Quality Index (PSQI), Somatic and Psychological Health Report (SPHERE), and some other symptom scales. However, many problems, such as an incomplete TCM syndrome differentiation system and the lack of unified standards for evaluation indicators, still exist. Here, we reviewed the clinical research of TCM in the treatment of CFS in the past five years, intending to provide a reference for the further study of the use of TCM therapies on CFS.

## 4. Syndrome Differentiation Treatment of TCM for CFS

At present, most clinical research on TCM syndrome differentiation treatment of CFS is mainly conducted through RCT, which covers TCM decoctions, proprietary Chinese medicines, and experiential prescriptions issued by clinical Chinese physicians. There is also syndrome differentiation treatment for a specific population. According to the main symptoms, they can be classified into liver stagnation and spleen deficiency syndrome, both heart and spleen deficiency syndrome, and other syndromes.

The total effective rate mentioned in this section equals the markedly effective cases plus the effective cases divided by the sample size and then multiplied by one hundred percent. The modern medical diagnostic criteria are based on the CFS diagnostic criteria revised by the Centers for Disease Control (CDC) in 1994 [16]. The diagnostic criteria of TCM and the total effective rate refer to the criteria for disease and TCM syndrome evaluation established by the Ministry of Health of the *Guiding Principles for Clinical Research of New Chinese Medicines* and *Diagnosis of TCM Syndromes* [24, 25]. Clinical cure means that the clinical symptoms disappear completely or basically and that the patient can adapt to a normal social life and work rhythm. A significant effect means that the clinical symptoms and signs are significantly improved, the main symptoms and concurrent symptoms are decreased by more than or equal to 2/3, or the symptom score is reduced to 2/3. Effective means that the clinical symptoms and signs are significantly reduced, the main symptoms and concurrent symptoms decrease by 1/3 to 2/3, or the symptom score is reduced to 1/3. Ineffective means that the clinical symptoms and signs are not significantly improved or are worsened and that the main and concurrent symptoms decrease by less than or equal to 1/3 or the symptom score drops less than 1/3.

### 4.1. Decoctions, Proprietary Chinese Medicines, and Syndrome Differentiation

#### 4.1.1. Liver Stagnation and Spleen Deficiency Syndrome

Li [26] observed 68 patients with this syndrome treated with Buzhong Yiqi decoction and Xiao Chai Hu Tang. The control group used adenosine triphosphate tablets at 20 mg bid po, and the observation group took the decoction (herbal composition: Codonopsis 25 g, *Astragalus* 30 g, Bupleurum 15 g, Agrimony 25 g, Atractylodes macrocephala 20 g, Pinellia tuber 15 g, Poria cocos 20 g, Tulip 20 g, Platycodon 6 g, Tangerine Peel 15 g, Scutellaria baicalensis 12 g, Licorice 12 g, and Jujube 12 g) at 150 ml bid po. After 4 weeks, the total effective rate of the observation group (97.06%) was higher than that of the control group (85.29%). Liu et al. [27] observed 72 patients with this syndrome treated with Chaihu Ramulus Cinnamomi decoction, which was made in granules in this trial. The control group was given a placebo, and the observation group took granules (herbal composition: Bupleurum 12 g, Cinnamon 9 g, Codonopsis 9 g, Scutellaria 9 g, Pinellia 9 g, White peony 9 g, Licorice 9 g, Ginger 6 g, and Jujube 6 g) mixed at 12 g bid po. After 4 weeks, the total effective rate of the observation group (88.89%) was higher than that of the control group (36.11%). Shi [28] observed 160 patients with this syndrome treated with Xiaoyaosan decoction. The control group took vitamin B at 2# tid po and oryzanol at 20 mg tid po, and the observation group took the decoction (herbal composition: *Angelica sinensis* 10 g, Radix paeoniae alba 10 g, Bupleurum 6 g, Poria cocos 20 g, Atractylodes macrocephala 10 g, Roasted Licorice 6 g, Peppermint 6 g, Codonopsis 15 g, and Chinese yam 20 g) at 150 ml bid po. After 3 weeks, the total effective rate of the observation group (89.74%) was higher than that of the control group (68.29%).

#### 4.1.2. Both Heart and Spleen Deficiency Syndrome

Yang et al. [29] observed 80 patients with this syndrome treated with Guipi decoction. The control group took fluoxetine hydrochloride capsules at 30 mg qod po, and the observation group took the decoction (herbal composition: Astragalus membranaceus 30 g, Suan date seed 25 g, Codonopsis 15 g, Tuckahoe with pine 15 g, Longan 15 g, Atractylodes 15 g, Polygala 15 g, Angelica 15 g, Licorice 10 g, and Radix aucklandiae 7 g) at 150 ml bid po. After 12 weeks, the total effective rate of the observation group (85.0%) was higher than that of the control group (67.5%). Wu et al. [30] reported another trial of 86 patients with this syndrome treated with Guipi decoction. The control group took rehabilitation training by instructing patients to jog 2 times a day for 30 min each time, and the observation group took the decoction (herbal composition: Codonopsis 20 g, *Astragalus* 20 g, Longan 15 g, fried Jujube seed 15 g, Poria cocos 15 g, Atractylodes rhizome 10 g, Angelica 10 g, Rhizoma chuanxiong 10 g, White peony root 10 g, Bupleurum 10 g, Citrus aurantium 10 g, Turmeric 10 g, Radix aucklandiae 10 g, Polygala 10 g, Tangerine peel 10 g, Cohosh 10 g, and Licorice 6 g). After 8 weeks, the total effective rate of the observation group (95.35%) was higher than that of the control group (72.09%). Ding [31] also reported the Guipi decoction used in a 60-patient trial. The control group used the same rehabilitation training as listed above, but the decoction of the observation group was slightly different and was composed of *Astragalus* 30 g, Semen ziziphi spinosae 25 g, Tuckahoe with pine 15 g, Longan 15 g, Polygala 15 g, Angelica 15 g, Atractylodes15 g, Codonopsis 15 g, Radix aucklandiae 7 g, and Licorice 10 g. After 8 weeks, the total effective rate of the observation group (93.33%) was higher than that of the control group (70.00%).

#### 4.1.3. Other Syndromes

Sun et al. [32] observed Shugan Yiyang capsule in the treatment of 80 male patients with liver stagnation and kidney deficiency syndrome. The control group took paroxetine hydrochloride tablets at 20 mg qd po, and the observation group added the capsule at 0.75 g tid po on the basis of the control group. After 4 weeks, the total effective rate of the observation group (90.00%) was higher than that of the control group (70.00%). Lin et al. [33] observed Fatigue Relief decoction in the treatment of 100 patients with Qi and blood deficiency syndrome. The control group was given Ginseng pills of Jinri brand at 1 g bid po, and the observation group took the decoction (herbal composition: *Codonopsis* 15 g, Poria cocos 9 g, *Atractylodes macrocephala* 9 g, Radix paeoniae rubra 9 g, *Astragalus* 12 g, Herba Epimedii 9 g, Polygalaceae 9 g, *Alisma* 9 g, Divaricate Saposhnikovia root 9 g, *Achyranthes bidentata* 9 g, *Adenophora* 9 g, *Rehmannia* 15 g, Jujube 3 g, and Ginger 3 g). After 4 weeks, the total effective rate of the observation group (94.0%) was higher than that of the control group (48.0%). Li and Wang [34] observed a kind of powder made up of Ginseng and Poria cocos at a ratio of 5 : 3 in the treatment of 80 patients with heart-qi deficiency syndrome. The control group was given coenzyme Q10 at 10 mg tid po, and the observation group took the powder. After 4 weeks, the total effective rate of the observation group (92.5%) was higher than that of the control group (77.5%).

### 4.2. Experiential Prescription and Syndrome Differentiation

Kang et al. [35] summarized Professor Liangduo Jiang's clinical experience in the treatment of CFS and believed that Chaihu Ramulus Cinnamomi decoction could balance Qi and Xue, and Yin and Yang reconcile the inside and outside and operate the cardinal machine to achieve the purpose of treating CFS. Kang also reported a case of a 42-year-old female patient whose diagnosis was a depressive state and CFS. By giving Jiang's experiential prescription (herbal composition: Bupleurum 15 g, Ramulus Cinnamomi 20 g, Gypsum 45 g, Cornu Saigae Tataricae 1.2 g, Radix scutellariae 15 g, Rhizoma pinelliae 9 g, Fructus Aurantii 15 g, Stir-fried citrus aurantium 15 g, Angelica 30 g, Worms 9 g, Hedgehog skin 9 g, Red peony 15 g, White peony 15 g, Dendrobium 15 g, Northern Adenophora 15 g, Coptis 9 g, Evodia fructus 6 g, Dinanstar 15 g, Atractylodes 15 g, Plumula Nelumbinis 5 g, Concha haliotidis 30 g, Malt 30 g, Dried human placenta 15 g, and Fried Semen ziziphi spinosae 15 g) at 150 ml bid po., the patient recovered after 6 weeks of treatment and a later follow-up, and there was no recurrence. Zhang et al. [36] summarized the Chief Physician Caida Gao's clinical experience in the treatment of CFS, whose technique was using the method of nourishing the heart and kidney, replenishing Qi, and nourishing Yin with the homemade Shenqi decoction. Additionally, he highlighted a case of a 42-year-old male patient whose diagnosis was CFS, who was given the Shenqi decoction (herbal composition: Ginseng 10 g, Salvia 30 g, Schisandra 6 g, Ophiopogon 15 g, Rehmannia 24 g, Dogwood 12 g, Fried yam 12 g, Achyranthes 10 g, Bidentata 12 g, Eucommia 12 g, Chinese wolfberry 12 g, Cuscuta 12 g, Amomum villosum 5 g, Blighted wheat 30 g, and Poria cocos 10 g) at 150 ml bid po. After 8 weeks, the patient's symptoms were significantly improved, which was followed by a prescription for 1 week. Consolidation treatment symptoms were removed after follow-up. Du [37] observed the curative effect of homemade Yishen Buxue cream, based on his clinical experience, which is actually a kind of decoction, in the treatment of 104 patients with CFS. The control group was given vitamin C 0.1 g + multivitamin-B 0.2 g + oryzanol 20 mg + adenosine triphosphate 20 mg tid po, and the observation group was given the decoction (herbal composition: *Angelica sinensis* 10 g, Rehmannia glutinosa 15 g, White peony root 10 g, Rhizoma chuanxiong 10 g, Dodder 15 g, Epimedium 12 g, Psoralen 10 g, and Chinese wolfberry 10 g) at 150 ml bid po. After 6 weeks, the total effective rate of the observation group (90.74%) was higher than that of the control group (75.93%). Liu et al. [38] observed 60 patients with CFS treated with her Lingzhi Yishou pills. The control group took fluoxetine tablets at 20 mg qd po, and the observation group took pills (herbal composition: Fleece flower 30 g, Tortoise plastron glue 12 g, Ganoderma 10 g, *Astragalus* 30 g, Panax notoginseng powder 5 g, Acorus gramineus soland 5 g, and Aster 10 g) at 100 ml tid po. After 4 weeks, the total effective rate of the observation group (90%) was higher than that of the control group (80%). Ma et al. [39] observed 80 patients with CFS treated with modified Erxian decoction, based on his clinical experience. The control group was given vitamin B1 120 mg + oryzanol 20 mg + bailemian 4# tid po, and the observation group took the decoction (herbal composition: Herba epimedii 15 g, Curculigo 10 g, *Morinda officinalis* 10 g, *Astragalus* 30 g, Codonopsis 15 g, Angelica 10 g, Cohosh 6 g, Bupleurum 6 g, Cork 5 g, and Anemarrhena 10 g) at 120 ml bid po. After 8 weeks, the total effective rate of the observation group (90.0%) was higher than that of the control group (77.5%).

### 4.3. Specific Population and Syndrome Differentiation

Liu et al. [40] observed Jianpi Jieyu Xiaopi cream (a kind of solid decoction made from high-dose herbals) in the treatment of 120 young CFS patients. The control group took oryzanol 20 mg tid po + Guipi Pill at 1# bid po, and the observation group took the cream (herbal composition: *Astragalus* 150 g, Codonopsis 120 g, Atractylodes macrocephala 100 g, Angelica 100 g, Rhizoma chuanxiong 100 g, Poria cocos 150 g, Chinese yam 150 g, Achyranthes bidentata 100 g, Fried Jujube seed 150 g, Albizia acacia peel 100 g, Fleece flower vine 300 g, Longan 100 g, Red peony 100 g, White peony 100 g, Bupleurum 60 g, Turmeric 100 g, Woody 60 g, Tangerine peel 60 g, Chickens gizzard-membrane 100 g, Licorice 100 g, and Tortoise plastron glue 250 g) at 15 g bid po. After 12 weeks, the total effective rate of the observation group (92.5%) was higher than that of the control group (70.0%). Wang [41] reported 2 clinical cases of using the same cream to treat CFS in young people. One was 26 years old, and another was 35 years old. Both were females. Both patients had obvious relief of symptoms after 8 weeks of treatment. Upon the follow-up 4 weeks later, the condition was cured, and the prescription was considered to be clinically effective. Pan and Chen [42] observed 60 cases of menopausal women with CFS treated with a low-dose estrogen patch. The control group used a placebo, and the observation group used an estradiol (E2) patch (specification: each tablet contains E2 2.0 mg, from which the daily release of E2 into the blood is 25 *µ*g, lasting 7 days), which should be changed every 7 days, and dydrogesterone 5 mg qd po. After 4 weeks, the symptoms and laboratory indicators of the observation group improved more than did those of the control group. In this study, the medication time was relatively short, and the number of cases was small, so the results obtained were limited; thus, further research is needed. In a short period of time, the E2 patch is simple and safe to use and has fewer adverse drug reactions. It can significantly improve CFS symptoms and improve the quality of life, providing another choice for women who are not suitable for oral medication.

## 5. External Treatment of TCM for CFS

The external treatment methods of TCM mainly include moxibustion, acupuncture, tuina, cupping, and other external treatment methods. Due to the diverse characteristics of methods and schools of TCM external treatment, when designing controlled experiments, we can see control experiments not only with external treatment methods but also with internal medicines.

The diagnostic criteria of TCM mentioned in this section refer to the relevant content of *Acupuncture and Moxibustion* [43], *Differential Diagnosis of TCM Syndrome* [44], and *Encyclopedia of Chinese Tuina* [45]. The clinical symptom score refers to the Fatigue Evaluation Scale compiled in 1993 by the Psychological Medicine Research Office of King's College Hospital in the United Kingdom [46].

### 5.1. Moxibustion

Chen et al. [47] observed 60 CFS patients treated with Fuyang moxibustion. The control group used mild moxibustion, and the observation group used this method. Both groups used the same acupoints (Dazhui GV 14, Ganshu BL 18, Shenshu BL 23, and Pishu BL 20) and were treated for 25 min once every other day. After 8 weeks, the total effective rate of the observation group (90.00%) was higher than that of the control group (73.33%). Liang et al. [48] observed 70 CFS patients treated with herb-partitioned moxibustion (HPM) (herbal composition: Aconite, Radix Rehmanniae, *Atractylodes macrocephala*, and *Salvia miltiorrhiza*, with an unknown dose) at back-Shu points. The control group received oral oryzanol 10 mg tid po, vitamin B1 10 mg tid po, and vitamin B6 10 mg tid po, and the observation group added this method on the basis of the control group. Both groups were treated for 30 min once a day. After 4 weeks, the total effective rate of the observation group (91.4%) was higher than that of the control group (77.1%). Tian et al. [49] observed 72 CFS patients treated with moxibustion at Gaohuang BL 43. The control group used ordinary acupuncture, and the observation group adopted this method. Acupoints (Gaohuang BL 43, Qihai CV 6, and Zusanli ST 36) were selected in both groups. Both groups were treated for 30 min once a day. After 4 weeks, the total effective rate of the observation group (88.9%) was higher than that of the control group (72.2%). Ma et al. [50] observed 76 cases of smokeless moxibustion in the treatment of CFS patients. The control group used ordinary acupuncture, and the observation group used this method. Both groups took the same acupoints (Guanyuan BL 26 and Zusanli ST 36) and treated for 25 min once a day. After 6 weeks, the total effective rate of the observation group (89.5%) was higher than that of the control group (76.3%). Xu et al. [51] observed the treatment of 94 CFS patients with both Spleen and Kidney Yang Deficiency Syndrome by Du-Moxibustion. The control group was given acupuncture treatment. The main acupoints were Yintang GV 29, Taixi KI 3, Taichong LR 3, Shenmen HT 7, and Sanyinjiao SP 6. The observation group used Du-Moxibustion from Dazhui GV 14 to Yaoshu CV 2, and the ingredients of the Du-Moxibustion were Musk 5 g, Corydalis 9 g, Clove 4 g, Asarum 5 g, Borneol 3 g, Aconite 4 g, Pangolin 3 g, and Cinnamon 5 g, which were ground into a powder and given at 2 g per serving. Both groups were treated for 20 min a day. After 12 weeks, the total effective rate of the observation group (91.50%) was higher than that of the control group (63.80%). Luo et al. [52] observed herb-partitioned moxibustion to treat 90 CFS patients with both Spleen and Kidney Yang Deficiency Syndrome. The control group was treated with ordinary acupuncture in which the acupoints were Shangwan CV 13, Zhongwan CV 12, Xiawan CV 10, Guanyuan CV 4, Qihai CV 6, and bilateral Tianshu ST 25, Zusanli ST 36, and Sanyinjiao SP 6. The observation group added herb-partitioned moxibustion (herbal composition: Nutmeg 15 g, Psoralen 30 g, Schisandra 9 g, Evodia 9 g, Wolfberry 15 g, Cuscuta 15 g, Astragalus 20 g, Codonopsis 30 g, Atractylodes 15 g, and Borneol 5 g) on the basis of the control group. Both groups were treated for 25 min a day. After 4 weeks, the total effective rate of the observation group (91.11%) was higher than that of the control group (73.33%).

### 5.2. Acupuncture

Modern TCM methods for acupuncture of CFS can be divided into body acupuncture and other acupuncture methods, mainly including abdominal acupuncture, auricular acupuncture, and acupoint embedding.

#### 5.2.1. Body Acupuncture

Wei et al. [53] observed 92 patients with CFS treated with acupuncture in the meridian route. The control group received conventional acupuncture, and the observation group used this method. Both groups used the same acupoints (Pishu BL 20, Ganshu BL 18, Shenshu BL 23, Baihui GV 20, Guanyuan CV 4, Zusanli ST 36, and Sanyinjiao SP 6) and were treated for 30 min a day. After 2 weeks, the total effective rate of the observation group (93.48%) was higher than that of the control group (82.61%). Chen et al. [54] observed acupuncture at the back-shu points of five zang organs (Feishu BL 13, Xinshu BL 15, Ganshu BL 18, Pishu BL 20, and BL 23) for the treatment of 60 patients with CFS. The control group selected the back-shu points of five zang organs at a distance of approximately 1.5 to 2 cm outwards to place acupuncture, and the observation group selected the back-shu points of five zang organs. Both groups were treated for 20 min twice a week. After 4 weeks, the total effective rate of the observation group (86.67%) was higher than that of the control group (53.33%). Guo et al. [55] observed 60 cases of CFS patients treated with electroacupuncture at the back-shu points. The control group used ordinary acupuncture, and the observation group used electroacupuncture. Both groups used the same acupoints (Baihui GV 20, Sishencong EX-HN 1, Taiyang EX-HN 5, Shenmen HT 7, Qihai CV 6, Zusanli ST 36, Sanyinjiao SP 6, and Neiguan PC 6), but the observation group added Xinshu BL 15 and Ganshu BL 18 on this basis. Both groups were treated for 30 min once a week. After 3 weeks, the total effective rate of the observation group (96.70%) was higher than that of the control group (73.30%).

#### 5.2.2. Other Acupuncture Methods

Wang et al. [56] observed 59 cases of CFS patients with Qi and blood deficiency syndrome treated by special drug acupoint catgut embedding. Control group 1 used acupoint catgut embedding, and control group 2 used warmed needle moxibustion. The observation group used special drug acupoint catgut embedding, which was infiltrated with Astragalus membranaceus injection for 30 ml. The three groups took the same acupoints (Zusanli ST 36, Guanyuan CV 4, Qihai CV 6, and Zhongwan CV 12). The treatments were taken once every 2 days for approximately 20 minutes each time. After 4 weeks, the total effective rate of the observation group (100%) was higher than that of control group 1 (95.24%) and control group 2 (88.89%). Xu et al. [57] observed 120 CFS patients with Qi deficiency syndrome treated by auricular gold-needle therapy. Control group 1 used auricular acupoints pressure therapy, in which Cowherb seed was adopted on only one ear, and control group 2 took Buzhong Yiqi pills at 6 g bid po. The observation group received auricular gold-needle therapy, in which one ear was acupunctured with a gold-needle and the other side was pressed with a Cowherb seed. The auricular acupoints were Shen CO 10, Xin CO 15, Fei CO 14, and Pizhixia AT 4. Each time, the auricular acupoints were pressed for 1 minute and treated once a week. A healthy control group was set up as a baseline. After 12 weeks, the total effective rate of the observation group (90.0%) was higher than that of control group 1 (80.0%) and control group 2 (82.5%).

### 5.3. Cupping or Tuina

Shi [58] observed 60 cases of CFS patients treated with balanced cupping therapy. The control group was given routine nursing care and dietary guidance; however, the specific method is unknown. The observation group added this method on the same basis. Both groups used the same acupoints (Dazhui GV 14, Xinshu BL 15, Ganshu BL 18, Pishu BL 20, Feishu BL 13, Shenshu BL 23, Yaoyangguan GV 3, and Laogong PC 8) for 20 min twice a week. After 4 weeks, the total effective rate of the observation group (93.30%) was higher than that of the control group (80.00%). Xia et al. [59] observed 140 patients with CFS treated with pivotal tuina. The control group used ordinary tuina for 12 min a day, and the observation group used this method (including rolling, plucking, and pushing methods) to tuina along the Shaoyang Sanjiao Meridian of Hand, Shaoyang Gallbladder Meridian of Foot, Shaoyin Heart Meridian of Hand, and Shaoyin Kidney Meridian of Foot for 30 minutes a day. After 4 weeks, the total effective rate of the observation group (91.42%) was higher than that of the control group (77.14%). Shang et al. [60] treated 70 patients with CFS by tuina on the back. The control group took Buzhong Yiqi pills at 6 g tid po, and the observation group used this method (including kneading back, push back, point back, rub back, and beat back methods) for 25 min a day. After 3 weeks, the total effective rate of the observation group (88.57%) was higher than that of the control group (74.28%).

### 5.4. Other External Treatments

Li and Hou [61] observed 80 CFS patients treated with hot compresses on acupoints. The control group took Liuwei Dihuang Wan at 9 g bid po, and the observation group used this method (acupoints Guanyun CV 4, Qihai CV 6, and Dazhui GV 14) for 30 min a day. After 8 weeks, the total effective rate of the observation group (92.5%) was higher than that of the control group (72.5%). Liang [62] observed 60 cases of CFS patients treated with scraping in the back-shu functional zone. The control group received oryzanol and vitamin B1 each at 10 mg tid po. The observation group added this method on the basis of the control group (the back-shu functional zone includes the chest 1 spine from the protrusion to the lower edge of the sacral 4 spinous process and within 3 inches of the paraspine) for 25 min once a week. After 4 weeks, the total effective rate of the observation group (90.0%) was higher than that of the control group (76.7%).

## 6. Combined Therapy of TCM for CFS

The combination therapy of TCM includes two or more external treatments combined or syndrome differentiation combined with external treatments. The treatment of CFS with TCM combination therapy can often complement the weaknesses, highlight the curative effects, and reduce the side effects caused by a single treatment method. The difficulty of clinical trials is often that the primary and secondary treatments are confused during the experiment.

### 6.1. Combination of Two or More External Treatments

Li and Sun [63] evaluated the efficacy of acupuncture and moxibustion in the treatment of CFS through meta-analysis, and the results showed that acupuncture and moxibustion have a certain effect in the treatment of CFS. The combined use of acupuncture and moxibustion, electroacupuncture and acupoint injection, body acupuncture, and auricular acupuncture may have advantages over medicine alone. In terms of the combination of two or more external treatments, the current clinical research mode mostly uses acupuncture plus one or two other external treatments.

#### 6.1.1. Acupuncture Combined with Moxibustion

Wu [64] observed 100 cases of CFS patients treated with acupuncture combined with moxibustion. The control group had only acupuncture, and the observation group took moxibustion after acupuncture. Acupoints Feishu BL 13, Xinshu BL 15, Ganshu BL 18, Pishu BL 20, Shenshu BL 23, Zusanli ST 36, Sanyinjiao SP 6, Yinlingquan SP 9, Zhongwan CV 12, Xiawan CV 10, Guanyuan CV 4, and Qihai CV 6 were selected for both groups for 30 min qod. After 3 weeks, the total effective rate of the observation group (96.00%) was higher than that of the control group (82.00%). Wang et al. [65] observed acupuncture combined with moxibustion in the lower Dantian to treat 48 patients with the Yang deficiency syndrome of CFS. The control group was treated with only acupuncture, and the observation group was treated with this method. Both groups took the same acupoints (Qihai CV 6, Guanyuan CV 4, Tian Shu ST 25, Zusanli ST 36, Taixi KI 3, and Taichong LR 3) and added moxibustion after that (acupoints Guanyuan CV 4, Qihai CV 6, and Shenque CV 8). Both groups were treated for 30 min twice a week. After 8 weeks, the total effective rate of the observation group (87.50%) was higher than that of the control group (62.50%).

#### 6.1.2. Acupuncture or Moxibustion Combined with Tuina

Qi et al. [66] observed a kind of consciousness-restoring resuscitation acupuncture and moxibustion combined with chiropractic therapy to treat 39 patients with CFS. The acupoints were Neiguan PC 6, Shuigou GV 26, Sanyinjiao SP 6, Shenmen HT 7, Yintang GV 29, Baihui GV 20, Tanzhong SI 11, Zhongwan CV 12, Guanyuan CV 4, Zusanli ST 36, Hegu LI 4, and Taichong LR 3. Patients were treated for 30 min qd. After 6 weeks, the total effective rate reached 89.74%. Xu et al. [67] observed abdominal acupuncture and moxibustion combined with balanced cupping to treat 200 patients with CFS. The control group was treated with Buzhong Yiqi pills at 3 g tid. The observation group used this method on the basis of the control group. The observation group was treated at acupoints Zhongwan CV 12, Xiawan CV 10, Qihai CV 6, Zusanli ST 36, Guanyuan CV 4, Huaroumen ST 24, Wailing ST 26, Daheng SP 15, and Juque CV 14 3 times a week for 30 min each time. After 8 weeks, the total effective rate of the observation group (95.00%) was higher than that of the control group (77.00%). Fu et al. [68] observed 77 cases of CFS treated by acupuncture and tuina combined with drug therapy. The control group was given a bid treatment of Anshen Bunao oral liquid. The observation group used acupuncture (acupoints: Sishencong EX-HN 1, Shenmen HT 7, and Sanyinjiao SP 6) to add back tuina for 55 min qd. After 4 weeks, the total effective rate (92.3%) of the observation group was higher than that of the control group (84.2%).

### 6.2. Combination of Syndrome Differentiation and External Treatments

Lu et al. [69] observed abdominal acupuncture combined with proprietary Chinese medicines to treat 68 patients with CFS. The control group took Jiawei Xiaoyao oral liquid at 10 ml bid po, Liuwei Dihuang Wan at 6 g bid po, and Guipi pills at 9 g tid po. The observation group added abdominal acupuncture (acupoints: Zhongwan CV 12, Xiawan CV 10, Qihai CV 6, Guanyuan CV 4, Huaroumen ST 24, and Wailing ST 26) on the basis of the control group. Abdominal acupuncture took 30 min qd in the first week and qod in the second week. After 4 weeks, the total effective rate of the observation group (82.4%) was higher than that of the control group (70.6%). Jiang [70] observed 60 cases of CFS patients treated with Ginger-partitioned moxibustion combined with Bazhen decoction. The control group was treated with electroacupuncture (acupoints: Baihui GV 20, Yintang GV 29, Neiguan PC 6, Shenmen HT 7, Taichong LR 3, Zusanli ST 36, and Sanyinjiao SP 6) for 30 mins qd. The observation group used Ginger-partitioned moxibustion (acupoints: Zhongwan CV 12, Guanyuan CV 4, Qihai CV 6, Zusanli ST 36, and Neiguan PC 6) for 30 min qd and added Bazhen decoction (herb composition: Angelica 10 g, Ligusticum 5 g, White peony 8 g, Rehmannia glutinosa 15 g, Ginseng 3 g, Stir-fried Atractylodes 10 g, Poria cocos 8 g, and Roasted Licorice 5 g) at 150 ml bid po. After 3 weeks, the total effective rate of the observation group (93.3%) was higher than that of the control group (83.3%). Hua et al. [71] observed 120 cases of CFS patients with heart and spleen deficiency syndrome with TCM decoction combined with auricular acupuncture. The control group was treated with oryzanol plus vitamin B1 at 20 mg each tid po. The observation group used this method. The decoction consisted of Codonopsis 20 g, Jujube seed 20 g, Atractylodes 20 g, Tuckahoe 20 g, Longan 12 g, Bupleurum 12 g, Pinellia 12 g, Scutellaria baicalensis 12 g, Ginger 8 g, Polygala 6 g, and Jujube 5 g, which were taken at 200 ml bid po. The auricular acupoints were Shenmen TF 4, Pi CO 13, Xin CO 15, Pizhixia AT 4, Shen CO 10, Neifenmi CO 18, and Jiaogan AH 6 a, which were treated by pressing alternately on both ears for 30 min qd. After 6 weeks, the total effective rate of the observation group (91.66%) was higher than that of the control group (63.33%). Li [72] observed Guipi decoction plus Xiao Chai Hu Tang combined with auricular acupuncture to treat 84 CFS patients with both heart and spleen deficiency syndrome. The control group took oryzanol and vitamin B1 tablets at 20 mg each tid po. The observation group used this method. The compound decoction consisted of Atractylodes 20 g, Codonopsis 20 g, Jujube seed 20 g, Bupleurum 20 g, Scutellaria 20 g, Bupleurum 20 g, Pinellia 12 g, Ginger 8 g, Polygala 6 g, and Jujube 5 g, which were taken at 200 ml bid po. The auricular acupoints were Shenmen TF 4, Shen CO 10, Pizhixia AT 4, Xin CO 15, Pi CO 13, and Jiaogan AH 6 a, which were treated by pressing alternately on both ears for 30 min qd. After 6 weeks, the total effective rate of the observation group (90.48%) was higher than that of the control group (71.43%). Xing et al. [73] observed 80 CFS patients treated with Shenqi Zhencao decoction combined with gentle moxibustion at Guanyuan CV 4 acupoint. The control group only used gentle moxibustion at Guanyuan CV 4 acupoint for 30 min qd. The observation group added Shenqi Zhencao decoction (herbs composition: American Ginseng, *Astragalus*, *Ligustrum lucidum*, *Eclipta prostrata*, *Polygonum multiflorum*, *Schisandra*, Stir-fried Jujube seed, Lily, Turmeric, *Epimedium*, and *Anemarrhena*, with the specific doses not provided) at 250 ml bid po. After 4 weeks, the total effective rate of the observation group (77.45%) was higher than that of the control group (47.50%).

## 7. Summary

Currently, there are many methods for treating CFS with TCM, showing its advantages. On one hand, treatments according to syndrome differentiation are diverse and involve herbal decoctions and proprietary Chinese medicines. External treatments include moxibustion, acupuncture, tuina, cupping, and other treatments. Combination therapies include two or more external treatments combined and internal and external treatments combined, which are often better than monotherapies and can remedy deficiencies. However, the clinical manifestations of CFS are different. Although fatigue is the main manifestation, there are individual differences in the appearance of insomnia or muscle pain in some patients. Treatments according to syndrome differentiation can provide patients with individualized treatments. On the other hand, TCM often has the advantages of simplicity, effectiveness, convenience, and a lower cost.

However, there are still many problems in the treatment of CFS with TCM. For example, diagnostic criteria of syndrome differentiation, treatment methods, and efficacy evaluations have not yet reached a consensus. Some therapies are quite cumbersome or rare, but the efficacy is almost the same. Sometimes, combination therapy is taken, but the focus on primary and secondary treatments is not clear. Therefore, it is difficult to compare which therapy is better, which reduces the credibility of the studies. Among the databases that we examined, RCT experiments accounted for a large proportion, and the total effective rates of observation groups were often over 90% and were even as high as 100%. However, there was a lack of repeated experiments. The level of evidence is not good enough, and high-quality clinical trials are still needed, which remains the main contradiction in the treatment of CFS with TCM. In the process of searching the papers, we also found some folk-specific therapies, such as a classic medicated diet porridge using herbs (composition: Yam 10 g, Semen euryales 10 g, Leek seed 10 g, and Japonica rice 50 g), which can improve symptoms such as fatigue by taking 500 ml once every other day [74]. Another one, which involves head scraping combined with music therapy for 15 min qd 5 times a week, may achieve similar results [75]. Spinal conditioning (a tuina technique) combined with tea therapy (a teabag made from Tangerine peel 20 g, Mint 15 g, Wild Jujube seed 20 g, and Bupleurum 20 g) can activate the brain [76]. Baduanjin exercise combined with acupuncture may strengthen immunity to treat CFS [77]. All these therapies might have effects on CFS to some extent, but related studies are rare, research data are relatively scarce, and safety cannot be guaranteed. Therefore, the recommendations are as follows. (1) To standardize the consensus of TCM diagnosis and treatment of CFS, unified and standardized syndrome differentiation and efficacy evaluation system should be established. (2) Multicenter, large-sample RCT experiments on CFS should be conducted to provide proof of evidence-based medicine using TCM. (3) CFS model research and animal experiments should be carried out to provide scientific theories for verifying the efficacy of TCM therapies. (4) Clinical studies of folk-specific therapies should be conducted, emphasizing the safety and reliability of clinical trials to enrich the TCM studies on CFS.

## Data Availability

No data were used to support this study.
